# Harnessing Big Data for Communicable Tropical and Sub-Tropical Disorders: Implications From a Systematic Review of the Literature

**DOI:** 10.3389/fpubh.2018.00090

**Published:** 2018-03-21

**Authors:** Vincenza Gianfredi, Nicola Luigi Bragazzi, Daniele Nucci, Mariano Martini, Roberto Rosselli, Liliana Minelli, Massimo Moretti

**Affiliations:** ^1^Department of Experimental Medicine, Post Graduate School in Hygiene and Preventive Medicine, University of Perugia, Perugia, Italy; ^2^Department of Health Sciences (DISSAL), University of Genoa, Genoa, Italy; ^3^Digestive Endoscopy Unit, Veneto Institute of Oncology IOV-IRCCS, Padua, Italy; ^4^Section of History of Medicine and Ethics, Department of Health Sciences (DISSAL), University of Genoa, Genoa, Italy; ^5^Hygiene and Public Health Unit, Local Health Unit 3 of Genoa, Genoa, Italy; ^6^Department of Experimental Medicine, University of Perugia, Perugia, Italy; ^7^Department of Pharmaceutical Sciences, Unit of Public Health, University of Perugia, Perugia, Italy

**Keywords:** big data, Zika, Ebola, Chikungunya, West Nile virus, dengue, Mayaro virus, communicable tropical diseases

## Abstract

**Aim:**

According to the World Health Organization (WHO), communicable tropical and sub-tropical diseases occur solely, or mainly in the tropics, thriving in hot, and humid conditions. Some of these disorders termed as neglected tropical diseases are particularly overlooked. Communicable tropical/sub-tropical diseases represent a diverse group of communicable disorders occurring in 149 countries, favored by tropical and sub-tropical conditions, affecting more than one billion people and imposing a dramatic societal and economic burden.

**Methods:**

A systematic review of the extant scholarly literature was carried out, searching in PubMed/MEDLINE and Scopus. The search string used included proper keywords, like big data, nontraditional data sources, social media, social networks, infodemiology, infoveillance, novel data streams (NDS), digital epidemiology, digital behavior, Google Trends, Twitter, Facebook, YouTube, Instagram, Pinterest, Ebola, Zika, dengue, Chikungunya, Chagas, and the other neglected tropical diseases.

**Results:**

47 original, observational studies were included in the current systematic review: 1 focused on Chikungunya, 6 on dengue, 19 on Ebola, 2 on Malaria, 1 on Mayaro virus, 2 on West Nile virus, and 16 on Zika. Fifteen were dedicated on developing and validating forecasting techniques for real-time monitoring of neglected tropical diseases, while the remaining studies investigated public reaction to infectious outbreaks. Most studies explored a single nontraditional data source, with Twitter being the most exploited tool (25 studies).

**Conclusion:**

Even though some studies have shown the feasibility of utilizing NDS as an effective tool for predicting epidemic outbreaks and disseminating accurate, high-quality information concerning neglected tropical diseases, some gaps should be properly underlined. Out of the 47 articles included, only 7 were focusing on neglected tropical diseases, while all the other covered communicable tropical/sub-tropical diseases, and the main determinant of this unbalanced coverage seems to be the media impact and resonance. Furthermore, efforts in integrating diverse NDS should be made. As such, taking into account these limitations, further research in the field is needed.

## Introduction

According to the World Health Organization (WHO), communicable tropical and sub-tropical diseases “occur solely, or principally, in the tropics” and “thrive in hot, humid conditions.” While some of these disorders, such as malaria, receive adequate treatment and research funding, other infections termed as “neglected tropical diseases” are relatively overlooked (1). Communicable tropical/sub-tropical diseases represent a diverse group of communicable disorders occurring in 149 countries, favored by tropical and sub-tropical conditions, affecting more than one billion people and imposing a dramatic societal and economic burden (2). Moreover, problems related to communicable tropical diseases control are mainly due to (i) tropical climate, that favors the spread of these diseases and (ii) the poverty of the regions affected by these diseases (1). Among the tropical/sub-tropical diseases there are also neglected tropical diseases that include a subset of 17 infectious disorders (caused by viruses, such as dengue, Chikungunya, and rabies, by prokaryotic organisms, such as Buruli ulcer, leprosy, trachoma, treponematoses, or by eukaryotic organisms, like Chagas disease, human African trypanosomiasis, leishmaniases, dracunculiasis, lymphatic filariasis, onchocerciasis, cysticercosis/teniasis, echinococcosis, foodborne trematodiases, and schistosomiasis) (3).

In the contemporary globalized society, the emergence/re-emergence of old and new infectious diseases, due to rapid human development in terms of demographics, populations, and environment, represent a serious public health concern (3). Communicable tropical diseases generate a relevant burden that disproportionately impacts on the world’s poorest, constituting, as such, a major barrier to development efforts in order to alleviate poverty and improve human health status and condition in the developing areas. Malaria and neglected tropical diseases kill more than 800,000 people annually and create long-term disability in millions more (4).

For communicable tropical disorders, the WHO, together with the United Nations Children’s Fund, the United Nations Development Programme, and the World Bank, has launched a “Special Programme for Research and Training in Tropical Diseases” (TDR), which represents a global program of scientific collaborations (1). Furthermore, the WHO has defined a Road Map for controlling and eliminating neglected tropical diseases by 2020 and has suggested some steps, which are fundamental in order to achieve these ambitious goals, including improved diagnostics, treatment strategy, and surveillance systems (5, 6).

Within the era of e-health, characterized by the diffusion of the new information and communication technologies (ICTs), non conventional, or novel data streams (NDS), such as web searches generated data or social media updates, are emerging as a new promising approach in enhancing/complementing traditional surveillance systems (7, 8) and/or supporting public health decision making (9). Actually, Big data, despite their promises and their potential, should not be considered or utilized as a substitute for traditional data sources, but, rather, as a valuable complementary approach. Algorithms and computational techniques they are built and rely on still need to be carefully refined, tuned, and calibrated, in order to avoid the risk of overfitting in Big data inference. For instance, this happened with “Google Flu Trend” (GFT), which failed to provide accurate predictions concerning influenza-like-illness (ILI) cases. GFT predicted, indeed, more than double the proportion of doctor visits for ILI than the centers for disease control and prevention (CDC) (10). Due to these concerns, GFT decided to no longer publish influenza estimates. Similarly, Google Dengue Trends, a web-based tool for predicting dengue cases, is not currently available.

**Figure 1:**
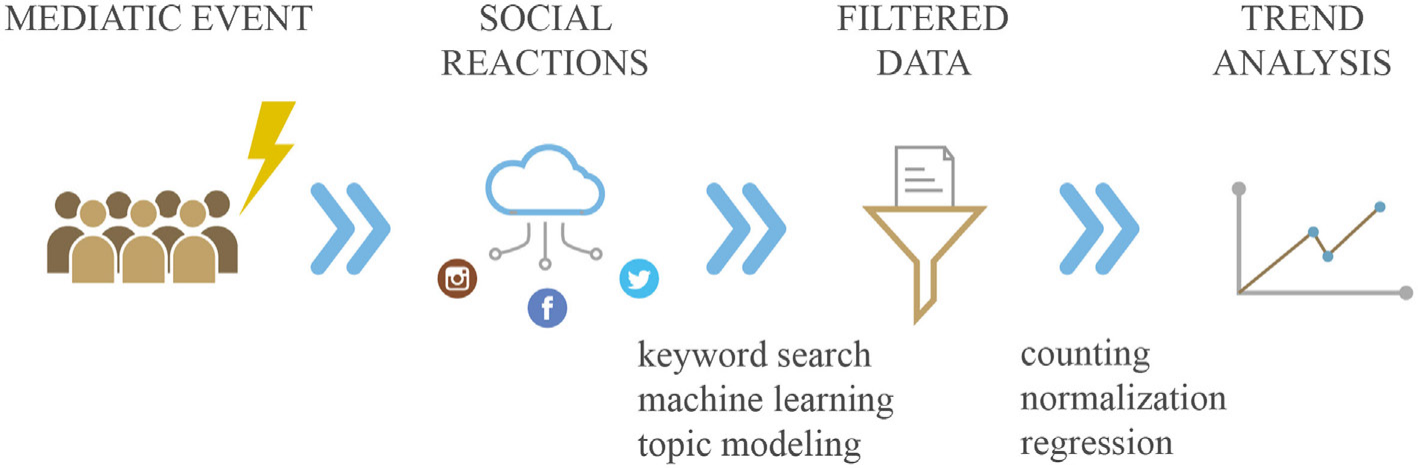
Collection and analysis of health-related demand data generated by novel data streams. Example of media event influencing society and consequent social reactions caught by social networks. Analysis of these data is represented by trend report.

“Infodemiology” (a *port-manteau* of information and epidemiology) and “infoveillance” (a combination of information and surveillance) have been coined by Gunther Eysenbach to indicate the new emerging “science of distribution and determinants of information in an electronic medium, specifically the Internet, or in a population, with the ultimate aim to inform and improve public health and public policy” (11). Systematically tracking and monitoring, collecting and analyzing health-related demand data generated by NDS could have the potential to predict events relevant for public health purposes, such as epidemic outbreaks, as well as to investigate the effect of media coverage in terms of potential distortions, misinformation and biases—the so-called “epidemics of fear” (12). Details are shown in Figure 1.

The aim of the current investigation was to systematically assess the feasibility of exploiting NDS for surveillance purposes and/or their potential for capturing public reaction to epidemic outbreaks. The main characteristics of NDS analyzed in this paper are briefly overviewed in Box 1.

Box 1 **Main characteristics of novel data streams (NDS) analyzed.**

**Table:** 

Novel data stream sources	Main characteristics
Google Trends	Online tracking system of Internet hit-search volumes

Twitter	Social network website, which allows users to publish short messages, visible to others

Youtube	Video-hosting website that allows members to store and serve video content

Baidu	Main Chinese Internet search engine company

HealthMap	Automated electronic information system for monitoring, organizing, and visualizing reports of global disease outbreaks according to geography, time, and infectious disease agent

Facebook	Social network website, where people can create own profiles and share information

Instagram	Social network website used to take and share photos

Google News	News aggregator provided by Google

Pinterest	Online service that allows to share images through social network

Sina Weibo	Most popular social media sites in China

Sina Micro	Popular Chinese social media that promotes websites, services, and products to promote collaboration within an organization

Wikipedia	Large website that provides free information

## Materials and Methods

The following systematic review was conducted according to the “Preferred Reporting Items for Systematic Reviews and Meta-Analyses” (PRISMA) guidelines (13). The literature search was performed in July–August 2017 using pre-established *ad hoc* key words and updated in September–October 2017. The search strategy is detailed in Table 1.

**Table 1:** Search strategy: inclusion/exclusion criteria, keywords, and filter applied.

Search strategy item	Search strategy details
Scholarly databases searched	PubMed/MEDLINE, Scopus, ISI/Web of Science

Used string of keywords	(computational model OR mathematical model OR big data OR infodemiology OR infoveillance OR digital epidemiology OR computational epidemiology OR NDS OR healthmap OR pinterest OR Instagram OR facebook OR google plus OR YouTube OR baidu OR sina micro OR twitter OR tweets OR microblog OR microblogging OR myspace OR blogs OR vlogs OR webinars OR forum OR Wikipedia OR wikis OR wikitrends OR social media OR social network) AND ("neglected tropical diseases" OR dengue OR chikungunya OR ebola OR zika OR malaria OR west nile OR leishmania OR leprosy OR hansen’s disease OR buruli ulcer OR echinococcosis OR Chagas OR teniasis OR cysticercosis OR trachoma OR lymphatic filariasis OR mycetoma OR chromoblastomycosis OR deep mycosis)

Time filter	None (from inception)

Language filter	None (any language)

Inclusion criteria	Primary original articles addressing the usage of non conventional data approaches for neglected tropical diseases

Exclusion criteria	Review articles or primary articles lacing quantitative details or presented at congresses or conferences and published as posters or in proceedings/gray literature

Target journals	Am J Infect Control, Asian Pac J Trop Med, BMJ, Disaster Med Public Health Prep, Epidemiol Infect, Health Commun, Health Informatics J, Health Secur, Int J Environ Res Public Health, Int J Infect Dis, J Am Med Inform Assoc, J Health Commun, J Med Internet Res, JMIR Public Health Surveill, J Public Health Manag Pract, Lancet, Lancet Glob Health, PLoS Negl Trop Dis, PLoS One, Public Health, Sci Rep, Springerplus, Travel Med Infect Dis

### Inclusion and Exclusion Criteria

Articles were included in the present systematic review whether they met the following inclusion criteria: (i) full text available; (ii) original articles; (iii) focused on communicable tropical and sub-tropical disorders, including neglected tropical diseases; and (iv) assessing novel sources of data, such as Twitter, web searches monitoring tools like Google Trends, Facebook, Google Plus, Wikipedia access logs, and traffic tracking tools, such as WikiTrends, and so on.

Exclusion criteria were: (i) studies without original data (abstract, letters to editor, editorials, comments, commentaries, expert opinions, reviews) and (ii) studies published in congress proceedings and gray literature.

No time and language filter was applied. Two researchers (NLB and VG), independently, screened title and abstract in order to verify the articles relevance. Possible disagreements were resolved through discussion or third reviewer consultation. The full text was downloaded only for the selected titles, and reference lists of included studies were also checked in order to identify any other potential relevant paper.

### Data Extraction

Main information, from the included studies, were extracted independently from two authors (VG and NLB) and collected in a pre-defined *ad hoc* spreadsheet. The collected data included: (i) surname of the first author, (ii) year of publication, (iii) data source, (iv) studied disease, (v) study period, (vi) location searched, (vii) used keywords, (viii) aim of the study, and (ix) main findings.

## Results

**Figure 2:**
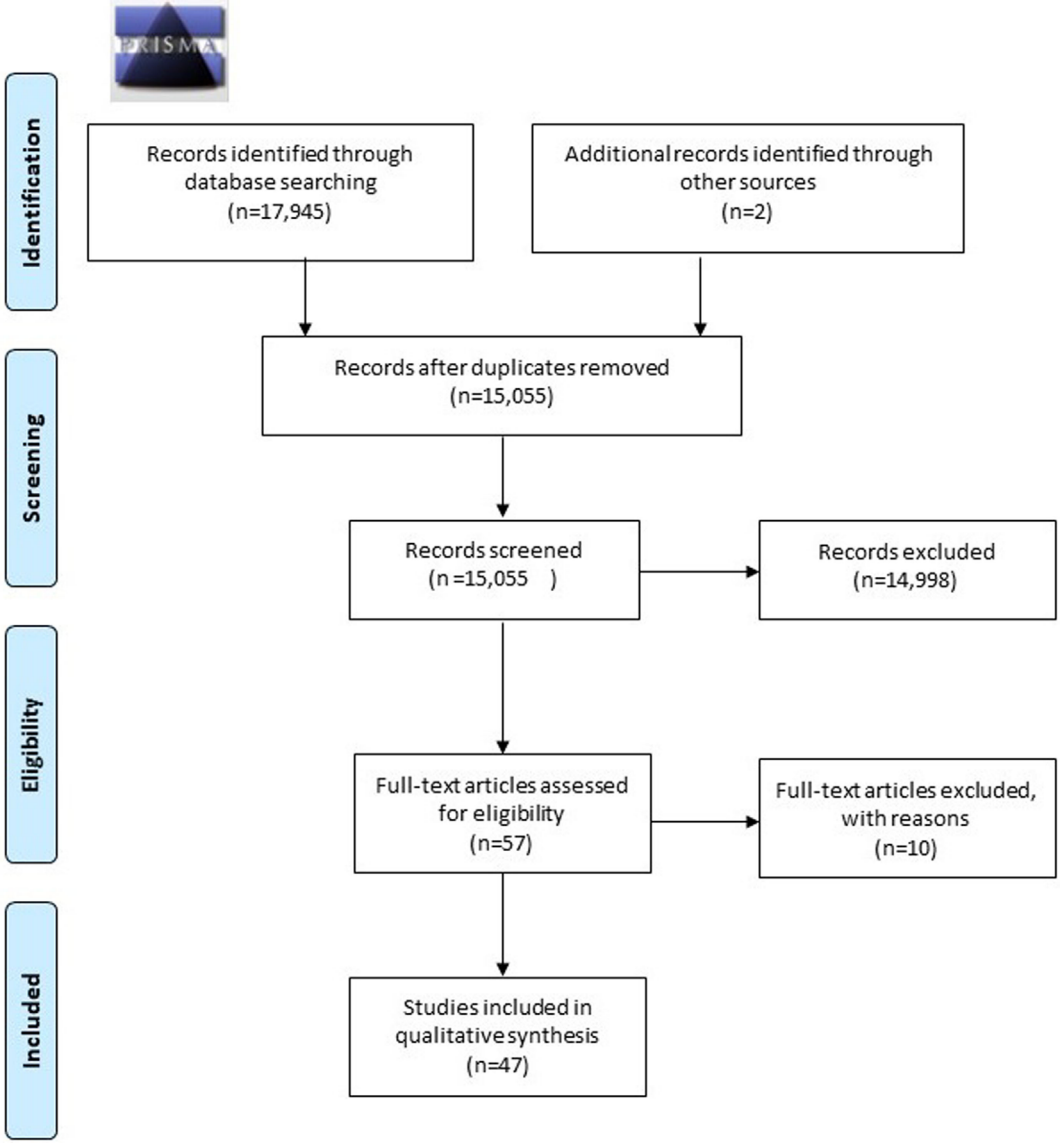
Flow diagram with screening process, according to the preferred reporting items for systematic reviews and meta-analyses guidelines.

A total of 17,945 articles were retrieved: two articles were found by means of extensive manual hand-searching and cross-referencing. After a preliminary screening, a total of 14,996 articles were excluded because they did not meet the inclusion criteria. Two more articles were retrieved from additional sources, finally 57 remaining articles were analyzed in full. 10 of them were excluded with reasons and last 47 articles were included in the present systematic review. Results are syntetized in (Table 2). The screening process is shown in Figure 2.

**Table 2:** Characteristics of studies included in the current systematic review.

Reference	Data source	Studied disease	Study period	Location searched	Purpose of the study	Used keywords	Type of analysis	Main findings
Roche et al. (14)	Twitter (423 tweets)	Chikungunya	The first 9 months of the 2014 outbreak	Martinique	To determine the predictive power of Chikungunya-related tweets	Chick*, Chik*	Correlational and regression analysis with epidemiological and environmental variables	Models integrating information from Twitter well explain epidemiological dynamics over time

Marques-Toledo et al. (15)	Twitter, Wikipedia access logs	Dengue	September, 2012–October, 2016	Brazil	To explore the predictive power of tweets in forecasting dengue cases	Dengue	Mathematical model	Tweets can be used to predict and forecast dengue cases

Nsoesie et al. (16)	Twitter	Dengue	Not applicable	Brazil	To understand the determinants of sharing tweets related to dengue	Dengue	Machine learning techniques	Sociodemographic variables play a major role in sharing dengue-related tweets

Ghosh et al. (17)	Websites reporting news	Dengue	2013–2014	India, China	To explore the predictive power of models incorporating news	Dengue	Mathematical models and time series-regression techniques	News-based models well correlated with epidemiological cases

Gomide et al. (18)	Twitter	Dengue	2006–April 2011	Brazil	To explore whether social media can be effectively integrated into disease surveillance practice	Dengue	Content analysis, correlational analysis and spatiotemporal analysis	Excellent correlation between tweets production and epidemiological cases (*R*^2^ = 0.9578)

Guo et al. (19)	Baidu	Dengue	January 2011–December 2014	China	To explore the feasibility of Baidu in real-time monitoring of Dengue	Dengue	Correlational analysis with epidemiological cases	A strong correlation was found

Li et al. (20)	Baidu	Dengue	Not applicable	China	To explore the predictive power of Baidu for forecasting Dengue cases	Dengue	Mathematical model	Baidu-based forecasting with one-week lag well correlated with epidemiological cases

Nagpal et al. (21)	YouTube	Ebola	Not applicable	Not applicable	To characterize the content of Ebola popular YouTube videos	Ebola	Content analysis	The most relevant YouTube videos were those presenting clinical symptoms

Strekalova (22)	Official centers for disease control and prevention (CDC) Facebook page	Ebola	18 March 2014–31 October 2014	Not applicable	To characterize the usage of new media from the CDC	Ebola	Content analysis	Audience engagement with Ebola posts was significantly higher compared to other non-Ebola topics, submitted by CDC

Odlum and Yoon (23)	Twitter (42,236 tweets—16,499 unique and 25,737 retweets)	Ebola	24 July 2014–1 August 2014	Not applicable (tweets in English)	To exploit Twitter as a real-time method of Ebola outbreak surveillance to monitor information spread	Ebola, #Ebola, #EbolaOutbreak, #EbolaVirus, and #EbolaFacts	Content analysis using NLP (Notepad++ and Weka) and correlation with epidemiological cases	Tweets started to rise in Nigeria 3–7 days prior to the official announcement of the first probable Ebola case

Pathak et al. (24)	YouTube (118 videos out of 198 videos)	Ebola	From inception–1 November 2014	Not applicable	To characterize Ebola-related YouTube videos	Ebola outbreak	Content analysis	The majority of the internet videos were characterized as useful, even though some videos were misleading

Roberts et al. (25)	English language websites and Twitter	Ebola	1 July 2014–17 November 2014	Not applicable	To qualitatively analyze the Ebola-related narrative	Ebola	Content analysis and sentiment analysis	Public engagement was directed toward stories about risks of U.S. domestic infections than toward stories focused infections in West Africa

Sastry and Lovari (26)	Official CDC and World Health Organization pages	Ebola	1 July 2014–15 October 2014	Not applicable	To understand the development of an ontological Ebola narrative	Ebola	Narrative analysis framework	Three themes: (a) consulting and containment, (b) international concern, (c) possibility of an epidemic in the United States

Liu et al. (27)	Baidu, Sina Micro	Ebola	20 July 2014–4 September in 2014	China	To understand the public reaction to the Ebola outbreak	Ebola	Mathematical model	Monitoring of social media enables to capture the spreading of fears related to epidemics outbreaks

Househ (28)	Twitter (2,592,5152 tweets) and Google News Trend	Ebola	30 September 2014–29 October 2014	Not applicable	To understand the role of the media coverage on public reaction to the Ebola outbreak in terms of digital activities	Ebola	Correlational analysis	A significant correlation between media coverage and tweets production was found

Jin et al. (29)	Twitter	Ebola	Late September 2014–late October 2014	Not applicable	To understand the public reaction to misinformation related to Ebola outbreak	Ebola or #ebola, #EbolaVirus, #EbolaOutbreak, #EbolaWatch, #EbolaEthics, #EbolaChat, #nursesfightebola, #ebolafacts, #StopEbola, #FightingEbola, and #UHCRevolution	Geo-coded analysis, coding, and mathematical model	Some rumors were more popular than others

Lazard et al. (30)	Twitter (2,155 tweets)	Ebola	2 October 2014	United States	To understand the public reaction to the Ebola outbreak	Ebola, #CDCcha	Content analysis using SAS Text Miner 12.1	Public concerned was about symptoms and lifespan of the virus, disease transfer and contraction, safe travel, and protection of one’s body

Alicino et al. (31)	Google Trends	Ebola	29 December 2013–14 June 2015	Worldwide	Real-time monitoring and tracking of Ebola virus outbreaks	Ebola, virus Ebola, Ebola virus, Ebola 2014, 2014 West Africa Ebola outbreak	Correlational and regression analysis with epidemiological cases	Correlation was stronger at a global level, but weaker at nation/country level

Basch et al. (32)	YouTube (100 most viewed videos viewed more than 73 million times)	Ebola	Not applicable	Not applicable	To analyze the most viewed Ebola-related videos	Ebola	Content analysis	YouTube could on the one hand enhance education and on the other hand spread misinformation

Fung et al. (33)	Twitter, Google Trends	Ebola	September 2014–November 2014	Worldwide	To understand the public reaction to the Ebola outbreak and the first US case	Ebola	Qualitative	Worldwide traffic on Twitter and Google increased as news spread about the first US case

Fung et al. (34)	Sina Weibo, Twitter	Ebola	8–9 August 2014 with a follow-up 7 days later	Not applicable	To capture the reaction to misinformation related to Ebola emergency	Ebola	Content analysis (manual coding)	Misinformation about Ebola was circulated at a very low level globally in social media

Wong et al. (35)	Twitter (1,648 tweets)	Ebola	September 2014–2 November 2014	United States	To understand the determinants of tweeting from local health departments	Ebola	Content analysis (manual coding from 2 independent authors) and regression analysis	Approximately 60% of local health departments sent tweets

Wong et al. (35)	Twitter *via* ArcGIS 10.2.2 and Google Trends	Ebola	September 2014–November 2014	United States	To understand the determinants of tweeting from local health departments	Ebola	Geospatial analysis	Weak, negative, non-significant correlation between online search activity, and per capita number of local health department Ebola tweets by state

Towers et al. (36)	Twitter (250,723 tweets), web searches	Ebola	29 September 2014–31 October 2014	United States	To understand the impact of the media coverage on the public reaction to Ebola outbreak	Ebola	Mathematical model	65–76% of the variance in samples was described by the news media contagion model

van Lent et al. (37)	Twitter (4,500 tweets from a corpus of 185,253 tweets)	Ebola	22 March 2014–31 October 2014	The Netherlands	To understand the predictors of Ebola-related tweet production	Ebola, #Ebola	Content analysis	Significant positive relation between proximity and fear

Strekalova (22)	Official CDC Facebook page *via* a Microsoft Excel add-on, Power Query	Ebola	25 March 2014–31 October 2014	Not applicable	To understand the usage of social media by the CDC	Ebola	Content analysis	Differences in audience information behaviors in response to an emerging pandemic, and health promotion posts

Fung et al. (38, 39)	Twitter (3,640 tweets on malaria)	Malaria	Not applicable	Not applicable	To characterize malaria-related tweets	#GlobalHealth, #malaria	Content analysis (with unsupervised machine learning techniques)	The main topics were prevention, control, treatment, followed by advocacy, epidemiology, and social impact

Ocampo et al. (40)	Google Trends	Malaria	2005–2009	Thailand	To exploit the predictive power of Google Trends in forecasting malaria cases	Malaria and malaria-related terms	Correlational analysis	Google Trends-based model well correlated with epidemiological cases

Adawi et al. (41)	Google Trends	Mayaro Virus	From inception (1 January 2004 on)	Worldwide	Real-time monitoring and tracking of Mayaro virus outbreaks	Virus Mayaro, Mayaro virus, virus de Mayaro, virus del Mayaro	Correlational and regression analysis	Web searches were driven by media coverage rather than reflecting real epidemiological cases

Bragazzi et al. (42)	Google Trends	West Nile virus	From inception (2004 on)	Italy	To exploit the predictive power of Google Trends	West Nile virus	Correlational analysis with epidemiological cases	A positive significant correlation between web searches and cases was found

Watad et al. (43)	Google Trends	West Nile virus	From inception (from 2004 on)	United States	To explore the predictive power of Google Trends	West Nile virus	Correlational and regression analyses and mathematical model	Good correlation between web searches and real-world epidemiological figures. Using data 2004–2015 it was possible to predict data for 2016

Basch et al. (44)	YouTube (100 most popular videos)	Zika	Not applicable	Not applicable	To analyze the most viewed Zika-related videos	Zika	Content analysis	Majority of YouTube videos concerned babies, cases in Latin American and in Africa

Bragazzi et al. (45)	Google Trends, Google News, Twitter, YouTube, and Wikipedia	Zika	1 January 2004–31 October 2016	Not applicable	To capture the public reaction to the Zika outbreak	Zika	Correlational and regression analyses	Public interest was constantly increasing, with public alert on teratogenicity of the Zika virus

Dredze et al. (46)	Twitter (138,513 tweets)	Zika	1 January 2016–29April 2016	Not applicable	To characterize Zika vaccine-related tweets	Zika vaccine	Content analysis (supervised machine learning techniques)	Most tweets contained misleading information

Fu et al. (47)	Twitter (1,076,477,185 tweets collected with Twitris 2.0 *via* API)	Zika	1 May 2015–2 April 2016	Worldwide	Content analysis of Zika-related Twitters data	Zika	Topic modeling was used to group bags of words. The 20-topic model was found to fit the data best, them were grouped in 5 themes	5 themes: (1) private/public response to the outbreak; (2) transmission routes; (3) societal impacts of the outbreak; (4) case reports; (5) pregnancy and microcephaly

Fung et al. (38, 39)	Pinterest (616 posts), Instagram (616 photos)	Zika	Not applicable	Not applicable	To characterize the Zika-related only material shared via Pinterest and Instagram	Zika virus, #zikavirus	Content analysis (manual coding)	Main languages were Spanish or Portuguese. Most popular topics were: prevention, pregnancy, and Zia-related deaths

Glowacki et al. (48)	Twitter 1,174 tweets collected	Zika	During an hour-long live CDC Twitter chat on February 12, 2016	CDC-generated tweets	Content analysis of Zika-related Twitters data	Zika	Text analytics to identify topics and extract meanings, using SAS Text Miner version 12.1	10 topics: virology, spread, infants’ sequelaes, how to participate to the chat, prevention, zika test, pregnants’ concerns, sexual transmission, encouraging to engage the chat, symptoms

Lehnert et al. (49)	913 obstetric practice websites randomly selected, Twitter and Facebook	Zika	January 2016–August 2016	Not applicable	To understand the determinants of social media usage from obstetric community	Zika	Regression analysis	25–35% of websites reported Zika-related information. Information *via* social decreased throughout time

Majumder et al. (50)	HealthMap and Google Trends	Zika	31 May 2015–16 April 2016	Colombia	To develop near real-time estimates for R_0_ and R_obs_ associated with Zika	Zika	Incidence Decay and Exponential Adjustment (IDEA) model to estimate R_0_ and the discount factor (d) associated with the ongoing outbreak	R_obs_ estimated with digital data is comparable with the number calculated with the traditional method

McGough et al. (51)	Google Trends, Twitter, HealthMap	Zika	May 2015–January 2016	Colombia, Venezuela, Martinique, Honduras, El Salvador	To explore the predictive power of non conventional surveillance techniques	Zika	Mathematical model	Integrating different non conventional surveillance techniques can improve prediction of Zika cases

Miller et al. (52)	Twitter (1,234,605 tweets collected with Twitris 2.0 *via* API)	Zika	24 February 2016–27 April 2016	Not applicable	To determine the relevancy of the tweets regarding: symptoms, transmission, prevention, and treatment	Zika, Zika virus, Zika treatment, Zika virus treatment	Content analysis with a combination of NLP and ML—annotation performed by three microbiologists and immunologists, supervised classification techniques, including J48, MNB, Bayes Net, SMO, SVM, Adaboost, Bagging, and topical analysis with LDA	The majority of the tweets were related to transmission and prevention, and were characterized by a negative polarity

Seltzer et al. (53)	Instagram (342 pictures out of 500 tagged images)	Zika	May 2016–August 2016	Not applicable	To characterize Zika-related images	#zika	Content analysis	Most images conveyed negative feelings (such as fear and concerns) and majority of shared pictures contained misleading information

Sharma et al. (54)	Facebook (top 200 posts)	Zika	For a week starting from 21 June 2016	Not applicable	To characterize the content of Zika-related Facebook posts	Zika	Content analysis	The misleading posts were far more popular than the accurate posts

Southwell et al. (55)	Twitter	Zika	1 January 2016–29 February 2016	United States, Guatemala, and Brazil	To determine the role of the media coverage on tweets production	Zika	Correlational analysis	A significant relationship between media coverage and digital behaviors was found

Stefanidis et al. (56)	Twitter (6,249,626 tweets)	Zika	December 2015–March 2016	Not applicable	To characterize Zika-related tweets in terms of temporal variations of locations, actors, and concepts	Zika	Spatiotemporal analysis	The spatiotemporal analysis of Twitter contributions reflected the spread of interest in Zika from South to North America and then across the globe, with a prominent role played by the CDC and WHO

Teng et al. (57)	Google Trends	Zika	12 February 2016–20 October 2016	Not applicable	To explore the predictive power of Google Trends	Zika	Mathematical model and correlation with epidemiological cases	The best predictive model was autoregressive integrated moving average (0,1,3)

Vijaykumar et al. (58)	Facebook pages of the Ministry of Health and National Environmental Agency (NEA) pages (1057 posts of which 33 were Zika-related)	Zika	1 March 2015–19 September 2016	Singapore	To understand the differences in outreach patterns between the preparedness and response stages of an outbreak	Zika	Thematic analysis	Prevention-related posts as garnering the most likes, while update-related posts were most shared and commented upon

**Figure 3:**
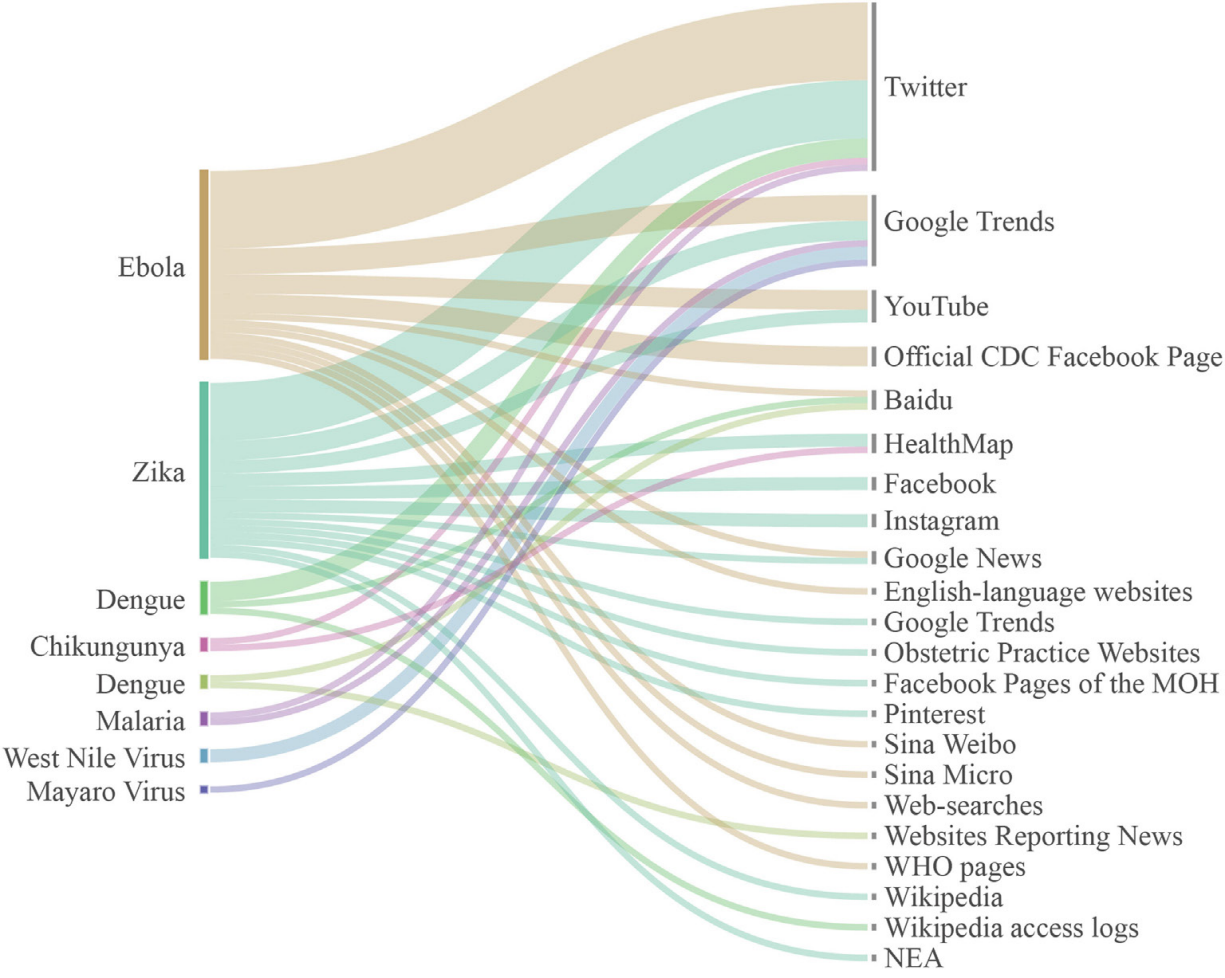
Communicable tropical/sub-tropical disorders and neglected tropical diseases investigated and non conventional sources analyzed, according to our results. Line thicknesses represent the volume of studies retrieved.

Out of 47 articles included in the review, 1 on Chikungunya, 6 on dengue, 19 were focused on Ebola, 2 on malaria, 1 on Mayaro virus, 2 on West Nile virus, and 16 on Zika. Looking at the non conventional data approaches used, 11 studies were searching on Google Trends, 5 on You tube, 2 on Wikipedia, 1 on Google News, 2 on HealthMap, 1 on Sina Weibo, and 1 on Sina Micro, 1 on Pinterest, 2 on Instagram, 3 on Web sites, 3 on Baidu, and 5 on Facebook. The most used data source was Twitter with 25 studies. However, some studies analyzed data on several sources, such as Fung and colleagues who search information about Zika virus on Pinterest and Instagram (39), or Househ et al. who searched on Twitter and Google Trends (28). Fifteen were dedicated on developing and validating forecasting techniques for real-time monitoring of neglected tropical diseases, while the remaining studies investigated public reaction to infectious outbreaks (Figure 3), in terms, for example, of sentiment analysis and spreading of fake news related to tropical disorder outbreaks.

### Neglected Tropical Diseases

#### Chikungunya Virus

Only one study was related to Chikungunya. Roche et al. (14) harnessed tweets related to Chikungunya posted during the outbreak in Martinique (14) and, performing a regression analysis with epidemiological and environmental variables, found that the integration of model and tweets contents well explained epidemiological dynamics over time.

#### Dengue

Five studies were related to dengue. Four of them relied on predictive models to predict dengue outbreaks. In Brazil, Gomide et al. (18) exploited Twitter and performed extensive content, correlation, and spatiotemporal analyses (18). Authors were able to find an excellent association between tweets production and epidemiological cases (*R*^2^ = 0.9578). Always in Brazil, Marques-Toledo et al. (15) utilized both Twitter and Wikipedia access logs in building predictive mathematical models for forecast dengue cases. In China, Guo et al. (19) leveraged Baidu for real-time monitoring and tracking of dengue cases (19). A strong correlation with epidemiological cases was found. In India and in China, Ghosh et al. (17) explored the predictive power of models incorporating websites reporting news related to dengue, carrying out mathematical models, and time series-regression techniques (17). News-based models were found to well correlate with epidemiological cases.

One article harnessed big data to explore the determinants of sharing tweets related to dengue. In particular, Nsoesie et al. (16), using machine learning techniques, found that sociodemographic variables played a major role in producing and sharing dengue-related tweets (16).

### Communicable Tropical/Subtropical Diseases

#### Ebola

Twenty articles were related to Ebola. All of them exploited big data sources to capture public reaction to Ebola outbreaks, both in terms of sentiments, fears, and concerns and of knowledge, beliefs, and attitudes. More in detail, four studies exploited Twitter. van Lent et al. (37) investigated the predictors of Ebola-related tweet production and found a significant positive relation between proximity and fear for Ebola virus (37). Jin et al. (29) harnessed Twitter to understand the public reaction to misinformation related to Ebola outbreak, performing an extensive geo-coded analysis, coding, and mathematical modeling (29). Authors found that some Ebola-related rumors were more popular than others Lazard et al. (30) found that the public was mainly concerned with symptoms and lifespan of the virus, disease transfer and contraction, safe travel, and protection of one’s body (30). Interestingly, Wong et al. (35) aimed at understanding the determinants of tweeting from local health departments. Approximately 60% of local health departments sent tweets (35).

Three studies utilized YouTube. Nagpal et al. (21) analyzed the most popular Ebola-related videos and found that the most relevant ones were those presenting clinical symptoms (21). Pathak et al. (24) found that the majority of the internet videos about Ebola were useful, even though some videos were misleading (24). Basch et al. (32) analyzed the 100 most viewed videos on YouTube with more than 73 million of visualizations and concluded that YouTube has a Yin–Yang nature, in that it could, on the one hand, enhance education and, on the other hand, spread misinformation (32).

Three studies utilized Facebook. Sastry and Lovari (26) analyzed the material posted on the official CDC and WHO pages (26). The following major themes were identified: (a) consulting and containment, (b) international concern, and (c) the possibility of an epidemic in the United States. Strekalova (22), reviewing the official CDC page, found that the CDC submitted fewer posts about Ebola than about non-Ebola topics, even though audience engagement was significantly higher (22). Furthermore, men were more interested in Ebola posts and submitted more comments per user. Moreover, Strekalova (22) found that there were differences in audience information behaviors in response to the emerging Ebola pandemic and health promotion posts (22).

Seven studies utilized more than one big data source. Fung et al. (33) used both Twitter and GT to understand the public reaction to the Ebola outbreak and the first US case (33). Fung et al. (34) combined Sina Weibo and Twitter to capture the reaction to misinformation related to Ebola emergency (34). Liu et al. (27) harnessed Baidu and Sina Micro to investigate the public reaction to the Ebola outbreak in China, performing a mathematical model (27). Roberts et al. (25) mined both English language websites and Twitter to qualitatively analyze the Ebola-related narrative, carrying out content and sentiment analysis (25). Househ (28), using Twitter and Google News Trend, found a significant correlation between media coverage and tweets production (28). Towers et al. (36) integrated Twitter and web searches to understand the impact of the media coverage on the public reaction to Ebola outbreak in the United States in terms of digital activities, performing a mathematical model (36). Wong et al. (35) exploited both Twitter and GT to understand the determinants of tweeting from local health departments Ebola, by means of a geospatial analysis (35). Authors found a weak, negative, non-significant correlation between online search activity and *per capita* number of local health department Ebola tweets by state.

Besides capturing public reaction to Ebola epidemic, three studies attempted to perform also predictive models and analyses. Alicino et al. (31) explored the feasibility of exploiting GT for a real-time monitoring and tracking of Ebola virus outbreaks, carrying out correlation and regression analysis with epidemiological cases (31). Authors found that correlation was stronger at a global level, but weaker at nation/country level, probably due to unbalanced, biased media coverage, and to digital divide. Odlum and Yoon (23) utilized Ebola-related tweets as a real-time method of Ebola outbreak surveillance to monitor information spread, capture early epidemic detection, as well as to examine content of public knowledge and attitudes (23). Authors found that tweets began to start to rise in Nigeria 3–7 days prior to the official announcement of the first probable Ebola case. Topics discussed included risk factors, prevention education, disease trends, and human compassion.

#### Malaria

GT was used for forecasting malaria cases by Ocampo et al. in 2013 (40). This study was performed using data related to Thailand in the period 2005–2009. Authors developed four Google search query-based models: namely, the so-called “microscopy model” (which uses terms associated with official data), the “automatic model” (based on automated selection algorithm), the “physician model” (generated from terms selected by surveyed Thai physicians), and the “stepwise model.” GT-based models well correlated with epidemiological cases.

Fung et al. (38, 39) used Twitter and performed a content analysis of the Malaria-related tweets (38). The main topics were: prevention, control, and treatment, followed by advocacy, epidemiological information, and societal impact.

#### Mayaro Virus

Only one study was related to Mayaro virus and exploited GT. Adawi et al. (41) explored the feasibility of utilizing GT for a real-time monitoring and tracking of Mayaro virus outbreaks (41). Correlational and regression analysis were performed with epidemiological cases and with other NDS, including Google News, PubMed/MEDLINE. Authors found that web searches were driven by media coverage rather than reflecting real epidemiological cases.

#### West Nile Virus

Two studies focused on the West Nile virus (42), and both of them used GT. Bragazzi et al. (42) aimed at exploiting the predictive power of GT (42) in Italy, performing a correlation analysis with epidemiological cases. Authors found a positive significant correlation between web searches and cases. Watad et al. (43) explored the predictive power of GT in the United States, carrying out correlation and regression analyses as well as mathematical modeling (43). Results showed a good correlation between web searches and real-world epidemiological figures. The best seasonal autoregressive integrated moving average model with explicative variable (SARIMAX) computed was (0,1,1)X(0,1,1)_4_, that is to say a “seasonal exponential smoothing” model. Moreover, using data from 2004 to 2015 it was possible to predict data for 2016.

#### Zika Virus

Sixteen studies focused on Zika and nine of them used Twitter as non conventional data source. In the majority of the cases (4 papers), the type of performed analysis was content analysis (46–48, 52), even though carried out with various research purposes. More in detail, Miller et al. (52) conducted a tweets analysis during the period of the hosting of the Olympics games and captured public reaction in terms of sentiments and concerns related to the potential association between Zika infection, microcephaly, and Guillain–Barrè syndrome, an association probable, but not yet confirmed at that time. Although the total polarity was negative, the percentage of positive tweets was higher than expected. An imbalance in the volume of tweets focusing on treatment was found. Similarly, a study by Fu et al. (47) lead to the emergence of five major themes: (1) government, private, and public sector, and general public response to the outbreak; (2) transmission routes; (3) societal impacts of the outbreak; (4) case reports; and (5) pregnancy and microcephaly. Glowacki et al. (48) investigated the use of new ICTs by healthcare authorities and organisms and, for the purpose, collected tweets during an hour-long live CDC Twitter chat, identifying 10 major topics. Some of them were related to the virology of Zika, spread, infants’, and pregnants’ sequelae, sexual transmission, and symptomatology. Dredze et al. (46) focused on the spreading of conspiracy theories and pseudo-scientific claims and found that tweets disseminating misleading information were concentrated almost all during the first week of pandemic (46).

Three studies used quantitative approaches, namely correlation and regression analysis (45, 49, 55), mathematical modeling (51), and spatiotemporal analysis (56). Southwell et al. (55) found strong positive correlations between news coverage, social media mentions, and online search behavior (55). Bragazzi et al. (45) found a constantly increasing public interest toward Zika, with the public opinion being particularly worried by the alert of teratogenicity of the Zika virus (45). In particular, the most frequent queries were about symptoms, transmission, and possible sequelae, such as microcephaly. Lehnert et al. (49) performed a regression analysis in order to understand the determinants of social media usage from obstetric community (49). The percentage of obstetric practice websites increased the number of information posted about Zika virus throughout the time, however, the proportion of practice sites posting Zika virus content on Facebook and Twitter declined. Practice websites related to university hospitals were more likely to post information on Zika virus compared to independent practice sites. McGough et al. (51) through a mathematical model, integrated different non conventional surveillance data (51), such as Google searches, Twitter microblogs, and the HealthMap digital surveillance system, and found that models relying on Google and Twitter showed the best 2- and 3-week ahead predictions. Last, Stefanidis et al. (56) performed a spatiotemporal analysis in order to characterize Zika-related tweets in terms of temporal variations of locations, actors, and concepts (56). The spatiotemporal analysis of the different Twitter contributions reflected the spread of interest in Zika from South America to North America and, then, across the globe. Healthcare institutional bodies, such as the CDC and the WHO, played a major role in tweet production.

Other type of big data sources explored in Zika studies were Facebook (54, 58), Google trends (50, 57), YouTube (44), Pinterest, and Instagram (39, 53). Vijaykumar et al. (58) analyzed the Facebook material posted on the public page of Ministry of Health (MOH) of Singapore and the Facebook page of National Environmental Agency (NEA), in order to evaluate the outreach and the engagement during the Zika pandemic. Generally speaking the MOH’s posts were more shared and received much more like compared to NEA’s post, however, the NEA’s posts were much more commented. Looking at the content, the NEA’s posts were more focused on prevention and intervention compared to the MOH’s posts with, in their turns, were more related to updates and investigations. Sharma et al. (54) analyzed the top 200 Facebook posts collected for 1 week starting from 21 June 2016 (54). The misleading posts were far more popular than the posts dispersing accurate, relevant public health information about the disease. Actually, the most popular relevant posts were published by the WHO, and obtained 43,000 views with 964 shares. The most popular misleading posts obtained, instead, more than 530,000 views, around 20,000 combined shares, and hundreds of comments.

Another big data source was GT. Actually, two studies examined GT-generated volume data in order to build predictive models. Teng et al. (57) aimed at predicting the number of infection cases (57). Authors constructed an autoregressive integrated moving average model (0, 1, 3) for the dynamic estimation of ZIKV outbreaks. Majumder et al. (50), using nontraditional digital data, such as HealthMap and Google Trends, tried to estimate the *R*_0_ and *R*_obs_ parameters of Zika virus spreading in Colombia. Authors observed an initially low, but increasing awareness and interest toward Zika. Google search was used in order to distribute more realistical over time, cumulative reported case counts. The ranges for *R*_obs_ estimated using digital data were well comparable with the figures calculated with the traditional method, even though a little lower. Transmission parameters can be estimated in real time using digital surveillance data, especially when traditional methods are not available.

Only one study assessed the content of YouTube videos on Zika (44). Basch et al. (44) analyzed the 100 most viewed English ZIKV-related videos. Among them, the majority were consumer-generated and Internet-based news videos. According to the contents, the majority of the videos concerned babies, cases in Latin American and in Africa.

Also Pinterest and Instagram were exploited, however, only two studies were conducted and both of them performed a content analysis (39, 53). Fung et al. (38, 39) analyzed more than 600 posts and photos on Facebook and Pinterest, respectively (39). The most popular topics were: prevention, pregnancy, and Zika-related deaths. Seltzer et al. (53) analyzed images posted on Instagram (53) and found that, even though the majority of posts focused on transmission and prevention, most of them conveyed negative feelings (such as fear and concerns) and contained misleading information.

## Discussion

In the past years, there has been a growing interest from the scholarly community in big data sources and their impact on public health. This was parallel to the interest toward neglected and communicable tropical diseases. Currently, communicable tropical diseases—including also the subset of neglected ones—represent re-emerging infections. However, re-emergence is not a completely new phenomenon occurring only in the past decades, actually it is happening since centuries. On the other hand, today re-emergence and dispersion of infectious agents are more rapid and geographically extensive, mainly due to globalization, and to arthropods or other vectors adaptation to its effects (59).

Novel data streams appear to be promising tools for predicting the spread of infectious agents, and, as such, can potentially aid and inform early decision support for when and how to employ public health interventions within a certain community. Emergency situations, being urgent scenarios, need accurate, reliable, and fast predictive models (60). Traditional surveillance systems are often plagued by a number of shortcomings and drawbacks, such as a significant delay in releasing official government-reported case counts (51). NDS seem to offer a real-time way to track and monitor outbreak dynamics, as well as to capture relevant information and parameters related to infection rates when these details are scarcely known or not available.

Novel data streams are also versatile tools in that they can be exploited to capture public reactions to epidemic outbreaks, in terms of emotion and fears, and of knowledge, attitudes, and practices. Some studies have harnessed big data sources to understand the spread of misinformation. Years of researches in the field of health communication and psychology have shown that opinion change represents a much more challenging issue than opinion formation, since, once people believe something wrong or misleading, it is difficult to dissuade them from such rooted beliefs (46). With respect to this topic, some studies have shown that NDS have a Yin–Yang nature, being, on the one hand, useful resources for promoting health education and being, on the other hand, vehicles of potentially dangerous information and content. In the era of the “post-truth,” the dissemination of fake news, alleged claims, and not evidence-based rumors could have serious implications in terms of public health. Techniques of social bookmarking and the direct involvement of healthcare workers and practitioners (in producing health-related websites, posting and sharing online material, tweeting, chatting, and so on) could be useful strategies (61).

Stakeholders and health authorities should be aware of the new ICTs, in that they could usefully exploit Internet-based tools for collecting the concerns of public opinion and replying to them, re-ensuring, and disseminating accurate, high-quality information (45). However, some studies included in the current systematic reviews have stressed gaps in usage of NDS by official healthcare organisms and bodies. Efforts should be made to convey a proper and effective health communication, utilizing ICTs and borrowing approaches from social marketing, making their posted material and delivered information more appealing, in terms of public outreach and engagement.

Another important point that should be stressed is that the value of each paper included in the current systematic review does not appear equal with respect to the field of public health. For example, the studies by Gomide and co-workers, McGough and collaborators, Odlum and Yoon, Roche and co-workers, and Teng and coauthors are highly relevant to public health outcomes (14, 18, 23, 43, 51, 57), while the others relate primarily to social networks. As such, only few papers with respect to the overall number of articles included in the present systematic review are directly relevant for public health outcomes. This definitely deserves further investigation and research in the field.

Our systematic review has some major strength, including the breadth of the search performed. However, even though efforts have been made in order to ensure completeness of the findings, alternate spellings/misspellings of keywords could have affected the results [for example, there are nine articles returned for “chikugunya” (an incorrect spelling of the disease chikungunya) returned recently on PubMed/MEDLINE]. On the other hand, reference lists of included articles have been extensively hand-searched, to increase the chance of getting all potentially relevant studies Relatedly, a variety of computational, “big data”-related terms (such as machine learning, collective intelligence or deep learning) were not included. *Ad hoc* search strings are, of course, finite in length, however, we expect to have included all relevant investigations meeting with inclusion/exclusion criteria on the basis that we have carried out extensive cross-referencing and additional hand-searching.

## Conclusion

Even though some studies have shown the feasibility of utilizing NDS as an effective tool for predicting epidemic outbreaks and disseminating accurate, high-quality information concerning communicable tropical diseases, some gaps should be properly underlined. Actually, among 47 studies included in our systematic review, only 7 studies focused on neglected tropical diseases (Chikungunya and dengue), while all the others were focusing on communicable tropical diseases (19 on Ebola, 2 on Malaria, 1 on Mayaro virus, 2 on West Nile virus, and 16 on Zika). In particular, out of the 17 groups of neglected tropical diseases individuated by the WHO, only two types of infectious diseases (namely, dengue and Chikungunya) were covered, and the main determinant of this unbalanced coverage seems to be the media impact and resonance, as well as the fear of the spreading of epidemic agents to Western countries. Furthermore, efforts in integrating diverse NDS should be made. As such, taking into account these limitations, further research in the field is needed.

## Ethical Statement

No ethical approval is required.

## Author Contributions

VG and NB contributed in conception and design of the study, data extraction and analysis. DN contributed in assembly and data interpretation. VG, NB, and DN drafted the manuscript. MM, RR, LM, and MM contributed in manuscript revision. All the authors approved the final version of the manuscript.

## Conflict of Interest Statement

The authors declare that the research was conducted in the absence of any commercial or financial relationships that could be construed as a potential conflict of interest.
